# An Online Platform to Provide Work and Study Support for Young People With Mental Health Challenges: Observational and Survey Study

**DOI:** 10.2196/21872

**Published:** 2021-02-09

**Authors:** Debra Rickwood, Vanessa Kennedy, Koki Miyazaki, Nic Telford, Stephen Carbone, Ella Hewitt, Carolyn Watts

**Affiliations:** 1 Faculty of Health University of Canberra Canberra Australia; 2 headspace National Youth Mental Health Foundation Melbourne Australia; 3 South East Melbourne Primary Health Network Melbourne Australia; 4 Prevention United Melbourne Australia

**Keywords:** youth, mental health, unemployment, work, study, online support

## Abstract

**Background:**

Young people, aged 15-25 years, are at a critical stage of life when they need to navigate vocational pathways and achieve work and study outcomes. Those with mental health problems are particularly at risk of disengagement with work and study and need effective support. The headspace Work and Study (hWS) service is an innovative online platform implemented in Australia to support young people aged 15-25 years with mental health problems to achieve work and study goals.

**Objective:**

This study aims to determine whether the hWS service has been implemented as planned, provides appropriate support for young people, and achieves its main goals.

**Methods:**

Data were collected via 2 methodologies: (1) the hWS Minimum Data Set, which includes data on all clients in the service (n=1139), services delivered, and service impact; and (2) a survey of hWS clients who volunteered to participate in an evaluation of the hWS service (n=137).

**Results:**

The service was accessed by its defined target group, young people aged 15-25 years with mental health and work and study difficulties. Young people found the online platform to be acceptable, and the assistance provided and clinical integration useful; many young people achieved positive work and study outcomes, particularly those who engaged more times with the service. More assistance was sought for work than study goals, suggesting that the transition to work may be particularly challenging for young people. One-third (298/881, 33.8%) of the sample for the service impact analyses achieved at least 1 primary work or study outcome, and this increased to 44.5% (225/506) for those who engaged with 5 or more sessions, demonstrating that greater engagement with the service produced better outcomes.

**Conclusions:**

Critical work and study support can be effectively delivered via an online modality to young people with common mental health problems. Digital services are scaleable to reach many young people and are of particular value for those with difficulty accessing in-person services.

## Introduction

### Background

In late adolescence and early adulthood, young people are at a critical stage of life vocationally; it is a time when they need to complete school, make career choices, navigate employment and training pathways, and move into the workforce [1]. Young people also have a heightened vulnerability to mental health problems [2] due to the major physical, emotional, and social changes that occur at this time of life. Many young people face significant barriers to vocational attainment and the emergence of mental health problems. As such, many young people need interventions that address both these critical needs.

Additional risk factors that can contribute to poor vocational outcomes at this stage of life include (1) structural, societal factors that affect what work is available for young people, as well as the nature of that work (especially with the impact of COVID-19); (2) a disadvantaged socio-economic background; (3) health problems or disabilities; (4) emotional or behavioral issues; (5) academic underachievement; (6) being homeless or having an unsuitable learning environment; (7) being an asylum-seeker or refugee; (8) having parental or caring responsibilities; (9) offending behavior; and (10) substance use issues [3]. Consequently, it is critical to focus on supporting young people at risk to assist them with effectively navigating work and study pathways during this vulnerable period using innovative approaches that are acceptable and effective.

Young people are uniquely at risk of disengaging from education, employment, and training. In Organisation for Economic Co-operation and Development (OECD) countries, an average of 6.3% of 15- to 19-year-olds and 15.1% of 20- to 24-year-olds were neither employed nor in education or training programs in 2018 [4]. For Australia, these figures were 5.3% and 11.9%, respectively. It is estimated that the unemployment rate for young Australians aged 15-24 years will reach 17% in the second half of 2020 [5] due to the impacts of the COVID-19 pandemic.

Disengaged young people are at increased risk of many negative outcomes, including social and economic exclusion, poor physical and mental health, reduced wellbeing, and involvement in antisocial behavior like criminal activity. Impacts can be long-lasting; young people who experience a period of disengagement can be at risk of “long-term socio-economic exclusion and getting caught in a ‘low-pay, no-pay cycle’ that can make it difficult to ever move out of poverty” [3]. In addition to being a potential consequence of disengagement, mental health difficulties can be a causal or correlating factor [6,7].

Mental health difficulties peak during adolescence and young adulthood, with approximately 1 in 4 young people impacted at some point [8]. Three-quarters of all mental health disorders first commence before the age of 24 years [9]. A recent study of young people who had accessed an Australian youth mental health service found that approximately one-third were not engaged in employment, education, or training [10], a proportion substantially higher than the general population. Importantly, the ability to obtain and maintain meaningful employment has been found to be one of the best indicators of recovery from mental health challenges [11].

As disengagement has major impacts, through lost taxes, public service costs, and costs associated with increased crime and poorer health, various policy and practice measures have been trialed and implemented to reduce young people’s disengagement across employment, education, and training [3]. There is strong evidence that integrating vocational interventions with mental health treatment leads to better outcomes for young adults with mental illness and that exposure to vocational interventions at an earlier age is associated with improved long-term employment outcomes [11].

The predominant intervention model that focuses on both mental health and vocational support is the Individual Placement and Support (IPS) model, for which international and Australian studies show strong supportive evidence [12-14]. Developed in the United States, the IPS approach is a vocational rehabilitation program designed to improve employment outcomes for people with severe mental illness. It has 8 practice principles: First, the goal is competitive employment (defined as employment that pays at least minimum wage, provides a commensurate salary with what others undertaking the same work receive, and is not exclusive to those with disabilities). Second, there is a zero-exclusion approach, whereby every individual with mental illness who wants to work is eligible for IPS assistance. Third, employment and mental health assistance are integrated. Fourth, client preferences—not the service providers’ preferences—drive the services provided and the types of jobs sought. Fifth, advice around benefits is provided. Sixth, job searching begins early on in a client’s engagement with IPS (commonly referred to as “rapid job search”). Seventh, IPS staff develop relationships with employers to ensure they are aware of and have a good understanding of job opportunities relevant to IPS clients. And eighth, time-unlimited, individual support is provided to clients, even after they gain work [15].

### The headspace Work and Study Service (hWS)

Launched in August 2016, the design of the hWS service was informed by the IPS model and its strong evidence-base. It is an initiative of headspace, Australia’s National Youth Mental Health Foundation. headspace was established in 2006 and represents the world’s largest national network of dedicated youth mental health centers [16]. headspace has a key focus on mental health support; however, vocational support is also a core service stream, alongside physical health and alcohol and other drug services [17].

The headspace center network's reach is augmented by eheadspace, an online and phone-based mental health care and information service that was launched nationally in 2011 [18,19], with information and support resources coordinated and distributed by the headspace national office. The main aim of headspace is to provide highly accessible, youth-friendly, early intervention and integrated, evidence-based support to young people aged 12-25 years through its centers, online services, and the national office [20].

Like IPS, the hWS service integrates employment and vocational services with clinical mental health and psychosocial support, focusing on the individual needs of people with mental health problems seeking to enter or remain in education or employment. The IPS model has been implemented mainly for people with serious mental health conditions (including low prevalence disorders, such as first-episode psychosis) rather than people with more common conditions (like depression and anxiety) and relies on direct, face-to-face client engagement. Unlike the IPS model, the hWS service operates predominantly for young people experiencing high-prevalence mental health issues (depression and anxiety) and offers personalized support from a work and study specialist via an online platform rather than in-person engagement. Online service delivery has the potential to overcome many service barriers, particularly for individuals who want support but also prefer some level of anonymity, live in hard-to-service rural and remote areas, or mistrust mainstream services. Online services are growing in popularity given most Australians (even isolated and marginalized groups) have internet access and utilize mobile devices in their daily lives [21]. Australia has a high level of internet penetration, estimated at 88% in 2018 [22]. Technology can be the preferred method of communication for some population groups, particularly young people, and has been given a major boost with the recent, sudden transition of many services to online modalities due to the COVID-19 pandemic.

The overarching goals of the hWS service are to increase the extent to which young people feel equipped to manage their work and study situation and increase the extent to which young people seek, attain, and maintain positive work or study outcomes. The core principles of the service are that it is (1) voluntary: unlike some employment agencies, young people are not required to use the service; (2) flexible: young people can make contact as much or as little as they want to during an episode of care, which is typically 3 months but can be longer; (3) personalized: the support young people receive is tailored to their needs and concentrates on the strengths young people have rather than those they do not have; (4) clinically integrated: hWS staff liaises with young people’s mental health providers to ensure work and study supports align with and reinforce their mental health interventions; (5) delivered with a no-exclusion approach: as long as young people are aged 15 to 25 years, experience mental health difficulties, and have work or study needs, they are eligible to use the service; and (6) accessible: no travel is required, operating hours are flexible, and the service is available via a range of online service delivery modes, such as synchronous chat, asynchronous email, telephone assistance, and video conferencing.

The hWS service is staffed by professionals with diverse experiences and backgrounds across the community, education, employment, and career support sectors. hWS staff provide young people with tailored one-on-one support via their preferred online modality. A typical episode of care with the hWS is up to 3 months of contact (approximately 1 full service per fortnight), during which time young people tend to work with the same professional. Support is offered for a period of up to 12 months after a work or study placement is achieved to align with evidence that employment interventions with long follow-ups are more likely to result in better employment outcomes for young people than those with short follow-up periods [11]. All hWS staff receive training in Mental Health First Aid and motivational interviewing given that the hWS service is designed to be appealing to and appropriate for young people with common mental health conditions, such as depression and anxiety. The service also has dedicated clinical staff who provide clinical governance over the hWS service, support hWS staff in providing clinically integrated care, provide clinical support directly to young people via the online platform, refer young people to other clinical services, and liaise with young people’s other clinical service providers.

### Our Study

This study aims to determine whether the hWS service has been implemented as planned, provides appropriate support for young people, and achieves its main goals. Specifically, we investigate whether (1) the service is utilized by its target group, namely young people experiencing both mental health and work and study difficulties; (2) young people find the service acceptable in terms of its service model and delivery via an online platform; (3) young people find the work and study assistance provided by the service useful; (4) young people find the service’s clinical integration helpful; and (5) young people achieve positive work and study outcomes during their time with the hWS service.

## Methods

### Design

To obtain data for the study, 2 methodologies were used: (1) the hWS Minimum Data Set (MDS), which provides data on all clients in the service, services delivered, and the service impact (ie, changes in the clients’ work or study status over time); and (2) a survey of hWS clients who volunteered to participate in an evaluation of the hWS. Ethics approval for the survey was obtained through the quality assurance approval process of the Melbourne Health Office for Research (QA2017146).

### hWS Minimum Data Set (MDS)

#### Participants

The hWS MDS provided information on 1139 hWS clients who received at least 1 hWS work or study session from August 9, 2016, to December 31, 2019. This sample is referred to as the MDS sample. Data for the analyses of the service impact were restricted to a subsample of 881 clients who received at least 2 work or study sessions (to enable measurement of a change in the work or study situation) and who did not have an open episode of care at the end of the reporting period. This sample is referred to as the MDS subsample.

#### Procedure

An online platform was used to collect all MDS data, including (1) client demographics, (2) client characteristics, (3) services provided, and (4) changes in a clients’ work or study situation. Data were recorded in the online MDS database by hWS staff at the time of first commencement with the service and at each occasion of service.

#### Measures

Client demographics included the age group the client belonged to at the time of first commencement with the service (15-17 years, 18-20 years, 21-23 years, or 24-25 years) and the client’s gender (female, male, another gender).

Client characteristics at the time of first commencement with the hWS service included psychological distress measured by the 10-item Kessler Psychological Distress Scale (K10) [23], which comprises 10 items that measure feelings of depression and anxiety over the past month on a scale that ranges from 10-50, with higher scores indicating greater distress. Distress scores were categorized as low (10-15), moderate (16-21), high (22-29), or very high (30-50) [24]. We also asked whether the client was currently receiving counseling from any other service (*yes*/*no*) and whether they were currently engaged in employment or training (*yes*/*no*).

Service provision measures included the number of work and study sessions clients received directly (hWS services such as client follow-up after a work or study placement, liaison with other care providers, case review, and care planning were excluded).

Service impact was measured by change in the clients’ work or study situation. At each session, staff could select from a detailed list of work or study changes that were coded into primary and secondary outcomes. Primary work outcomes were “gained work” (including apprenticeships and traineeships) and “gained a better job than the one they had” (ie, one that they enjoyed more, is more aligned with their studies, and had a higher salary). Primary study outcomes were “began study” and “swapped to a course/training that was better than what they were previously studying” (ie, something more likely to lead to good job outcomes and something they enjoyed more). Secondary work outcomes were “increased work hours,” “resolved job at risk,” “engaged in an Australian government employment service,” and “obtained a job or volunteer placement.” Secondary study outcomes were “increased study” and “resolved study at risk.”

### Survey

#### Participants

Of the 711 hWS clients asked to participate, 137 clients completed the survey, a response rate of 19.3%. This sample is referred to as the Survey sample.

#### Procedure

All clients who received support from the hWS service between August 9, 2016, and December 4, 2018, (n=711) were asked as part of their registration process whether they agreed to be contacted about future research and evaluation activities. SurveyMonkey software was used to email an invitation to complete the survey to clients who had agreed and had provided a valid email address. For eligible participants who received support prior to November 2017, the email invitation was sent in December 2017; for remaining eligible participants, the email invitation was sent in December 2018. If clients were in both samples and completed the survey twice (n=17), only their first completed survey was included in our analyses. Clients completed the survey online at their convenience.

#### Measures

The survey consisted of questions relating to the clients’ experiences with and perceptions of the service. The measurement domains relevant to this study were the (1) appropriateness of the hWS service model, (2) acceptability of the hWS service’s online service delivery model, (3) usefulness of the hWS service’s work and study assistance, and (4) usefulness of the hWS service’s clinical integration.

The appropriateness of the hWS service model was measured via 6 positive statements about the service with a 5-point Likert response scale (0=*strongly disagree* to 5=*strongly agree*). Example items were “The service is appropriate for people aged 15-24 years,” “I would recommend the Digital Work and Study service,” and “I was satisfied with the Digital Work and Study staff I had contact with.”

The acceptability of the hWS service’s online service delivery model was assessed by 2 items listing 5 potential benefits and 5 potential challenges of online delivery, for which respondents were permitted to select multiple responses for each item.

The usefulness of the hWS service’s work and study assistance was based on 8 positive statements about the service measured on a 5-point Likert response scale (0=*strongly disagree* to 5=*strongly agree*). Example items were “I feel supported in pursuing my work and study goals” and “I took steps to achieve my work and study goals.”

The usefulness of the hWS service’s clinical integration was determined by 5 positive statements about the service measured on a 5-point Likert response scale (0=*strongly disagree* to 5=*strongly agree*). An example item was “I feel supported in managing my mental health and wellbeing issues.”

Total scale scores were derived by averaging over all the items in each domain (with the exception of the acceptability measure, which was multi-response). These could range from 1-5, with higher scores indicating more positive views of the hWS service within the domain. All scales attained acceptable internal consistency: Cronbach alpha=.95, .95, and .92 for appropriateness, usefulness of assistance, and usefulness of clinical integration, respectively.

### Data Analysis

Data were analyzed using Business Intelligence software (version 2018.1; Tableau Software LLC), Excel (version 15.0.05301.1000; Microsoft), and SPSS (version 25.0; IBM Corp). Descriptive statistics were computed, and chi-square tests of homogeneity and independence were used to examine group differences (*P*<.05).

## Results

### Demographics and Characteristics

Table 1 presents the demographics and presenting characteristics for each sample group. Chi-square tests revealed that the 3 samples did not differ significantly on age, gender, K10 group, receipt of counseling at commencement, and the work and study situation at commencement.

**Table 1:** Demographics and presenting characteristics of study participants (n=1139) by sample group. [Due to missing data, columns may not sum to total n. Answering the 10-item Kessler Psychological Distress Scale (K10) was optional (8.3% chose not to). Other client demographic and characteristic measures were captured for the vast majority of clients, although occasionally, clients chose not to provide these responses.]

Demographic/presenting characteristics		MDS^a^ sample (n=1139), n (%)	MDS^a^ subsample (n=881), n (%)	Survey sample (n=137), n (%)
**Age in years**				
	15-17	137 (12.8)	104 (12.5)	13 (9.7)
	18-20	403 (37.6)	320 (38.3)	50 (37.3)
	21-23	389 (36.3)	295 (35.3)	49 (36.6)
	24-25	143 (13.3)	116 (13.9)	22 (16.4)
**Gender**				
	Female	624 (58.9)	494 (58.8)	77 (58.3)
	Male	391 (36.9)	315 (37.5)	48 (36.4)
	Another gender	45 (4.2)	31 (3.7)	7 (5.3)
**K10 category at the time of commencement**				
	Low	62 (5.9)	44 (5.5)	5 (4.1)
	Moderate	180 (17.2)	139 (17.3)	25 (20.3)
	High	349 (33.4)	270 (33.7)	47 (38.2)
	Very high	453 (43.4)	349 (43.5)	46 (37.4)
**Receiving counseling at the time of commencement**				
	Yes	717 (63.7)	551 (63.2)	85 (63.0)
	No	409 (36.3)	321 (36.8)	50 (37.0)
**Work/study situation at the time of commencement**				
	Not working or studying	480 (42.1)	373 (42.3)	67 (49.3)
	Working and/or studying	659 (57.9)	508 (57.7)	69 (50.7)

^a^MDS: Minimum Data Set

### Service Utilization by Target Group

The hWS service provided 1139 clients with 7897 work and study support sessions. The number of sessions per client ranged from 1 to 98, with an average of 6.9 (SD 7.7) and a median of 4.0 sessions. Of the 1139 clients, 47 received support over 2 episodes of care, and 2 received support over 3 episodes of care. As shown in Table 1, all clients were in the target age range of 15-25 years, with three-quarters aged 18-23 years, and a higher percentage of clients were women.

MDS data suggest the service reached young people experiencing both mental health and work and study difficulties. Table 1 shows that, of the valid responses, more than three-quarters (802/1044, 76.8%) of the clients presented with a K10 rating that indicated high or very high psychological distress, close to two-thirds (717/1126, 63.7%) were receiving counseling support at the time they presented to the hWS service, and close to half (480/1139, 42.1%) were not working or studying at the time they presented to the hWS service. In terms of reasons for accessing the hWS service, the majority (779/1139, 68.4%) sought work-related assistance, while 16.8% (191/1139) sought career planning assistance and 14.8% (169/1139) sought study-related assistance.

### Appropriateness of the Service Model and Usefulness of Assistance

Table 2 displays findings for 3 of the survey measure domains. For the appropriateness of the hWS service, the average score was well above the scale midpoint. The vast majority of respondents (87.8%) agreed overall that the hWS service model was appropriate. For individual scale items, agreement with the appropriateness of the service for young people aged 15-24 years and satisfaction with staff were particularly high. There were 85.8% (109/127) who agreed they would recommend the service, and 81.9% (104/127) agreed that the service was meeting their expectations.

For the domain of usefulness of the hWS assistance, once again, the average score was well above the scale midpoint. The majority of survey respondents (82.5%) agreed that the hWS service’s work and study assistance were useful. Satisfaction was particularly high to the extent to which the service helped respondents feel supported in pursuing, identifying, and taking steps toward achieving their work and study goals. More than three-quarters (101/132, 76.5%) of survey respondents indicated that the work and study assistance they received had helped them feel confident that they could manage their work and study situation in the future.

For the domain of usefulness of hWS clinical integration, most survey respondents (72.2%) agreed the hWS service’s clinical integration was useful. Satisfaction was particularly high with regard to whether the service helped respondents realize how their mental health and wellbeing impacted their work and study situations. Satisfaction was lower with regard to the extent to which the hWS service assisted young people to reduce the impact of their mental health and wellbeing issues on their lives more generally. The mean scores for the 3 survey measure domains all different significantly from each other (*P*<.001) with the lowest score for the domain of usefulness of hWS clinical integration.

**Table 2:** Survey domain percentages and means for the survey sample (n=137). (There were minimal missing data for the total scale scores: 2.2% for appropriateness, 5.1% for usefulness, and 7.3% for integration.)

Survey domain	Respondents in each response category					
	Strongly disagreed, %	Disagreed, %	Neither agreed nor disagreed, %	Agreed, %	Strongly agreed, %	Average score, mean (SD)
Appropriateness of hWS^a^ model	1.3	4.0	6.9	33.8	54.0	4.4 (0.8)
Usefulness of hWS^a^ service work/study assistance	1.4	6.6	9.5	38.7	43.8	4.2 (0.8)
Usefulness of hWS^a^ service clinical integration	0.7	9.4	17.7	39.8	32.4	4.0 (0.9)

^a^hWS: headspace Work and Study

### Value of Online Platform

The benefits and challenges of the online platform are presented in Table 3. The vast majority of respondents (115/129, 89.1%) experienced benefits, and close to two-thirds (80/129, 62.0%) of respondents did not experience any challenges with online service delivery. Most respondents indicated that they found it beneficial that no travel was required and that online service delivery was less confronting than in-person service delivery. In terms of challenges, just over one-fifth (28/129, 21.7%) indicated that they would have preferred in-person support, and less than one fifth (19/129, 14.7%) indicated that they experienced some difficulty in explaining their situation via an online platform.

**Table 3:** Potential benefits and challenges of online service delivery reported by the survey sample.

Benefits and challenges of digital service delivery		Respondents, n (%)
**Benefits (n=129)**		
	No travel was required.	110 (85.3)
	It was less confronting than meeting in-person.	63 (48.8)
	If in-person support was the only option, I may not have sought help.	30 (23.3)
	I live in an area with limited services; access to a national service was helpful.	27 (20.9)
	Other benefit(s)	9 (7.0)
	Indicated that none of the listed benefits applied	14 (10.9)
**Challenges (n=129)**		
	I would’ve preferred to meet in-person with someone.	28 (21.7)
	I found it difficult to explain my situation via the digital platform.	19 (14.7)
	I found it difficult to get through to the Service on the phone.	11 (8.5)
	I found the Digital Work and Study Service/eheadspace website difficult to use.	9 (7.0)
	Other challenge(s)	6 (4.7)
	Indicated that none of the listed challenges applied	80 (62.0)

### Effectiveness at Helping Young People Achieve Positive Work and Study Outcomes

Table 4 shows that more than one-third (298/881, 33.8%) of the MDS subsample achieved a primary work or study outcome, and close to half (396/881, 44.9%) achieved a primary or secondary outcome. For those who received at least 5 work or study hWS sessions, just under half (225/506, 44.5%) achieved at least 1 primary outcome, and more than half (289/506, 57.1%) achieved a primary or secondary outcome. There was no significant difference according to gender (*P*=.409), but there was a significant age difference (*X*^2^_1_=6.2; *P*=.013); just over a quarter (49/187, 26.2%) of those under 19 years of age, compared to over one-third (233/648, 36.0%) of those at least 19 years of age, achieved at least 1 primary outcome. There was a significant association between the number of sessions received and achieving both a primary (*X*^2^_4_=94.2; *P*<.001) and primary or secondary (*X*^2^_4_=109.0; *P*<.001) outcome, with strong linear trends showing that the likelihood of achieving outcomes increased with more sessions.

Across the 3 broad reasons that clients access hWS support (work, study, career planning), trends were similar, with no significant difference (*P*=.678) in the percentage of clients achieving a primary outcome, with about one-third overall achieving a primary outcome. There was, however, a significant difference in the percentage of clients who achieved either a primary or secondary outcome (*X*^2^_2_=8.3; *P*=.016), with those seeking study-related support significantly more likely to achieve an outcome compared to those seeking work-related assistance. Those who were not working or studying when commencing the hWS service were significantly more likely to achieve a primary outcome (*X*^2^_2_=12.8; *P*<.001), reaching 40.5% (151/373).

Table 4 provides an indication of the association between the total number of sessions received and the likelihood of a positive outcome being recorded. However, further analyses were undertaken to explore the average number of sessions clients received prior to a positive outcome first being recorded. These indicated that of those in the MDS subsample who gained a primary outcome and for whom appropriate data were available (n=297), the average number of sessions to achieve the first primary outcome was 8.4 (SD 7.16; range 1-44). Of those who gained a primary or secondary outcome and for whom data were available (n=395), the average number of sessions required to achieve the first outcome was 7.6 (SD 6.23; range 1-42).

**Table 4:** Demographics and characteristics of the Minimum Data Set subsample clients who achieved primary or secondary outcomes during their time with the headspace Work and Study (hWS) service (n=881).

Characteristic		Achieved at least 1 primary work or study outcome, n (%)	Achieved at least 1 primary or secondary work or study outcome, n (%)
Full sample (n=881)		298 (33.8)	396 (44.9)
**Age in years (n=835)**			
	<19 (n=187)	49 (26.2)	77 (41.2)
	≥19 (n=648)	233 (36.0)	297 (45.8)
**Gender (n=840)**			
	Male (n=315)	105 (33.3)	131 (41.6)
	Female (n=494)	167 (33.8)	232 (47.0)
	Another gender (n=31)	14 (45.2)	15 (48.4)
**Number of hWS sessions (n=881)**			
	2-3 (n=268)	44 (16.4)	62 (23.1)
	4-5 (n=175)	49 (28.0)	74 (42.3)
	6-7 (n=120)	37 (30.8)	53 (44.2)
	8-9 (n=87)	39 (44.8)	48 (55.2)
	≥10 (n=231)	129 (55.8)	159 (68.8)
Received the median number of hWS sessions (≥5; n=506)		225 (44.5)	289 (57.1)
**Primary goal in working with the hWS service (n=881)**			
	Work-related (n=590)	200 (33.9)	246 (41.7)
	Study-related (n=135)	49 (36.3)	73 (54.1)
	Career planning–related (n=156)	49 (31.4)	77 (49.4)
**Work/study status at commencement (n=881)**			
	Not working or studying (n=373)	151 (40.5)	163 (43.7)
	Working or studying (n=508)	147 (28.9)	233 (45.9)

## Discussion

### Principal Findings

The results revealed that the hWS service was achieving its main goals. It was being utilized by its defined target group of young people aged 15-25 years with both mental health and work and study difficulties. Young people found the online platform acceptable, and the assistance provided and clinical integration useful. Moreover, positive work and study outcomes were achieved by many young people, particularly those who engaged more with the service.

The vast majority of young people accessing the service had high or very high psychological distress and were engaged with mental health support services, and almost half were not working or studying at the commencement of the service. This shows that the service was being accessed by the target client group of young people with mental health issues who were disengaged or at risk of disengagement from work and study. Over the first 3 years of the service’s development and implementation, over 1000 young people received services. Most were aged 18 years and over (87.2%), revealing that the greatest need is for those in early adulthood transitioning from high school into work or tertiary education. A higher percentage of clients were women. Despite this gender disparity, the hWS service was more successful in reaching young men (36.9%) than eheadspace (the headspace online mental health service), in which less than 20% of clients are men. Australian men are particularly hard to reach in the online mental health environment [18]. Nevertheless, it would benefit the service to find better ways of reaching and providing more appropriate support for young men, who tend to be a harder-to-reach group for all mental health–related programs [25].

Overall, however, young people found the online platform to be acceptable. Most young people reported benefits of the online platform, and few reported challenges. The lack of the need to travel was by far the greatest benefit, but almost half (63/129, 48.8%) said that it was less confronting than meeting in person. There were, however, one-fifth (28/129, 21.7%) who reported that they would have preferred to meet in-person with someone, showing that there is still a preference for face-to-face contact for some young people. A growing body of research shows that while many people are adapting to and prefer online support options, there is still a considerable proportion who prefer in-person services [26]. The online platform is a major point of distinction of the hWS service from IPS models, which use a traditional face-to-face approach. Importantly, by using an entirely online model, the service can be made available to many more young people, including those who live in rural and remote areas or who have other barriers to accessing in-person services.

The hWS was seen to be very appropriate to the late adolescent and early adulthood age range, and the assistance provided and clinical integration of their mental health care were perceived as useful by most young people. Appropriateness to the age group and satisfaction with staff were particularly strong. Primarily positive responses were provided to all the items assessing appropriateness and usefulness. The lowest rated item was for the service reducing the impact of mental health and wellbeing issues on the young person’s life more generally. This reflects that while the service integrates mental health support, the service's main focus is on improving work and study engagement rather than more general life-satisfaction factors.

One-third (298/881, 33.8%) of the total sample achieved at least one primary work or study outcome, and this increased to 44.5% (225/506) for those who engaged with 5 or more sessions, demonstrating that greater engagement with the service produced better outcomes. There was a linear relationship of outcome achievement with engagement with more sessions. No comparative studies are available, as the hWS service is the first to provide work and study support to young people with common mental disorders using an online platform. A study of 146 Australian young people with first-episode psychosis implementing a traditional IPS model reported positive outcomes for 71% of clients engaged in the program for 6 months [27], but this program comprised considerably greater investment and time and was more intensive than the hWS service.

Much more assistance was provided for work than for study. This reveals a greater gap in the need for support for work. It is likely that young people are more aware of avenues for further study, as tertiary study institutions promote their courses and entry processes through open days, marketing, and online information. Tertiary education institutions generally provide significant support for commencing students. In contrast, the workplace is dispersed with multiple entry points to a diverse range of industries and settings that are not coordinated, and consequently, young people need more support to navigate the transition to work.

### Limitations

It is important to consider these findings in the context of the limitations of the study. Firstly, the study was observational and there was no comparator group. As such, causation cannot be ascertained. The questions in the survey were developed specifically for the hWS and their validity is not assured. There were also limited options for open-ended questions, which might have provided deeper insights into service satisfaction and impact. The survey was optional and had a modest response rate (albeit typical for such surveys); therefore, findings must be interpreted with caution as they may not reflect the opinions and experiences of other hWS clients. We also did not have information on the specific mental health problems and disorders that were being experienced by the young people accessing the hWS service, and so we cannot determine whether it was more or less acceptable and seen as effective for young people experiencing different types of mental health concerns.

Further, as Australia’s Aboriginal and Torres Strait Islander young people experience particularly high levels of disengagement, further research is needed to explore the extent to which the hWS service is reaching Aboriginal and Torres Strait Islander young people, as well as the extent to which the service is appropriate and effective for this population group. Additionally, as a goal of the hWS service is to increase the extent to which young people maintain work and study outcomes, further research should explore the extent to which work and study outcomes are maintained over time (which was outside the scope of this study). Ideally, a randomized controlled trial would be used to establish causality, and an economic evaluation would be undertaken to identify the potential value and long-term cost savings achieved by the service.

### Conclusions

The design of hWS was based on strong evidence from many years of research into IPS models, and findings from this study suggest the service is effective in achieving its aims to help young people attain important work and study outcomes and that the online model of the service is appropriate. Now that it is established, the service is potentially scalable to provide effective work and study support to more young people across the country at a relatively low cost. There is also potential for it to be adapted in other countries.

## Abbreviations

hWS: headspace Work and Study
IPS: Individual Placement and Support
K10: 10-item Kessler Psychological Distress Scale
MDS: Minimum Data Set
